# Relapse in class II orthognathic surgery: a systematic review

**DOI:** 10.1186/s12903-022-02636-x

**Published:** 2022-12-15

**Authors:** Stephanie Eckmüller, Eva Paddenberg, Karl-Anton Hiller, Peter Proff, Helge Knüttel, Christian Kirschneck

**Affiliations:** 1 Department of Orthodontics, University Medical Hospital Regensburg, Franz-Josef-Strauss-Allee 11, 93053 Regensburg, Germany; 2 Department of Operative Dentistry, University Medical Hospital Regensburg, Franz-Josef-Strauss-Allee 11, 93053 Regensburg, Germany; 3 University Library, University Medical Hospital Regensburg, Franz-Josef-Strauss-Allee 11, 93053 Regensburg, Germany

**Keywords:** Orthognathic surgery, Stability, Class II, Systematic review

## Abstract

**Objectives:**

Relapse after orthognathic surgery seems to depend on diverse factors. Proffit et al. postulated in 2007 a “hierarchy of stability” (Head Face Med 6:66, 2007), ranking posttreatment stability after various orthognathic procedures, but no systematically reviewed evidence was provided. Therefore, the aim of this review was to investigate the extent of class II relapse in orthognathic surgery of Angle class II patients depending on the surgical procedure used.

**Materials and methods:**

Seven databases were searched for randomized and controlled clinical trials to compare relapse in surgical procedures for Angle class II patients. After duplicate study selection, data extraction and risk of bias assessment were performed with the ROBINS-I tool as well as data synthesis by frequency distribution, followed by assessment of the quality of evidence with GRADE.

**Results:**

Four non-randomized cohort-studies with a total of 132 patients were included. Bimaxillary procedures as well mandibular advancement procedures proved to be highly stable. Single jaw interventions at the maxilla achieved mostly stable results at sagittal dimension and problematic stability in the vertical dimension. However, there were only limited data available with low quality of evidence.

**Conclusions:**

Limited existing evidence of low quality partly support the postulated hierarchy of stability of Proffit et al. (Head Face Med 6:66, 2007) and indicates that a surgical correction of class II dysgnathia with bimaxillary procedures and mandibular advancement seems to be highly stable. However, additional studies are needed to address the relation between relapse and surgical orthognathic intervention.

*Trial registration* PROSPERO 2019 CRD42019144873.

**Supplementary Information:**

The online version contains supplementary material available at 10.1186/s12903-022-02636-x.

## Introduction

Orthognathic surgery entails an interdisciplinary therapy between orthodontics and maxillofacial surgery for the treatment of patients with dysgnathia of a certain severity. Dysgnathia describes hereditary or acquired anomalies of jaw shape and position of maxilla and mandible. These can disrupt facial aesthetics as well as occlusion and articulation. The need for combined orthodontic and surgical therapy depends on a lot of variables. One criterion is an occlusion not adjustable by the orthodontist due to the skeletal situation. Further factors include the axial alignment of the dentition in relation to the jaw bases, the positioning of the dentition within the alveolar bone plus a proper tooth and jaw positioning in sagittal as well as in transversal direction. Orthognathic surgery involves long and invasive treatment for patients. Changes in facial aesthetics have to be expected or are even a reason for surgery. That is why treatment prognosis and limits have to be explained to the patients. Especially the relapse rate of the diverse interventions matters and can facilitate the decision, whether or not to undergo surgery [10, 13].

Relapse means that treatment outcome regresses towards the initial dysgnathia. The risk of relapse depends on diverse factors, such as the type of dysgnathia, operative procedure, extent and direction of the operative relocation, means of fixation, age of the patient and possible growth potential, incidence of remodelling and resorption, incidence of orthodontic recurrence and presence of an unsecured occlusion [10, 13]. Regarding the stability, we can deduce the prognosis for the different interventions, which Proffit et al. postulated 2007 in a “hierarchy of stability” [18]. A superior repositioning of the maxilla and advancement of the mandible are alleged to be highly stable (restricted to patients with a short and normal face height) as well as genioplasty in any dimension. Maxillary advancement and correction of asymmetry are reported to be stable. Bimaxillary interventions (maxilla up and mandible forward; maxilla forward and mandible back) and the correction of mandibular asymmetries are purported to be stable only with a rigid fixation. Three procedures are reputed to be rather problematic regarding post-operative stability: the isolated mandibular setback, a downward movement of the maxilla and a widening of the maxilla. These statements, however, are not yet validated. The question arises, whether the postulated hierarchy of stability postulated by Proffit et al. [18], especially in Angle Class II anomalies.

The present work aims to systematically review the literature and assess evidence from clinical studies on human patients about the relapse rate after different types of orthognathic surgery to correct Angle Class II anomalies.

## Materials and methods

### Protocol and methods

This review’s protocol was registered a priori in PROSPERO (CRD42019144873), and all post-hoc changes were appropriately noted. This review is conducted and reported according to the Cochrane Handbook [7] and PRISMA statement [11] (Additional file 1: Appendix 1).

### Eligibility criteria

Human Angle class II dysgnathia patients of any age, sex or ethnicity with potential relapse after orthodontic treatment involving orthognathic surgery were included. Participants with skeletal or dental class II were included, without requiring specific diagnostic criteria. Studies on patients undergoing orthognathic surgery due to syndromes, cleft lip and palate or trauma were excluded. No limitations concerning language, publication year or publication status were applied.

The primary outcome was the extent of relapse within at least 1 year after orthognathic surgery to correct Angle Class II anomalies, that is the loss of any correction of jaw position achieved by the treatment by comparing the position of the jaws after surgery with the position of the jaws after a defined time period in lateral X-rays (cephalograms) based on the change of angular and metric cephalometric parameters assessing the sagittal and vertical position of the upper and lower jaw. The relapse extent was categorized according to the scheme of Bailey et al. [2]: highly stable ≤ 10% chance of significant change; stable < 20% chance of significant change; problematic ≥ 20% chance of significant change. Therefore, each patient acted as his own control. Studies that assess the relapse rate only subjectively or qualitatively, but not measured this quantitatively by means of angular and metric cephalometric measurements of the sagittal and vertical position of the upper and lower jaw, were excluded, as these did not allow an objective evaluation of relapse.

### Information sources and search

A broad literature search was performed until July 16, 2019, in the databases EMBASE, MEDLINE, Cochrane Library, Web of Science, ClinicalTrials.gov, WHO`s International Clinical Trials Registry Platform, and Google Scholar. Primary search strategies for EMBASE and MEDLINE were developed and adapted for the other databases [9] (Additional file 1: Appendix 2). Relevant subject headings from the databases’ controlled vocabularies as well as a broad range of text words in order for sensitive searches were selected. Syntax to the search interfaces was adapted. Search strategies were developed by a medical librarian (HK) with input from the project team. In addition, the reference and citation lists of the eligible full text articles and of relevant systematic reviews were manually screened. In Science Citation Index Expanded (Web of Science) all works citing the seminal works of Proffit et al. [17] and Bailey et al. [2] were searched. No date limit was employed.

Randomized controlled trials (RCTs) and controlled clinical trials (CCTs) were considered in preference. Because not even three RCTs or CCTs with at least moderate risk of bias were found, comparative cohort studies and case–control studies were considered as well. Other study types were excluded, such as case-reports and case series, editorials, letters or replies, conference reports, comments, expert opinions, non-clinical studies, in-vitro studies, in silico studies, animal studies and studies with non-eligible outcomes.

### Study selection

Two reviewers (SE and EP) independently screened the titles or abstracts of studies retrieved using the search strategy and those from additional hand-searching to remove duplicates and to identify articles that potentially meet the inclusion criteria, without documenting the reasons for exclusion. Any differences between the two reviewers were settled by consensus after consulting a third author (CK). The full text of these potentially eligible studies, as well as of those abstracts, which did not provide sufficient information to allow decision-making as regards inclusion or exclusion, were retrieved and assessed by one review author (SE), while a second checked the decisions (EP). For each article excluded based on the screening of the full text, the reason for exclusion was documented (Additional file 1: Appendix 3). Any differences between the two reviewers were resolved by discussion with a third author (CK).

### Data collection process and items

For assessment of study quality and evidence synthesis, standardized, predefined forms were used to extract relevant data from included studies. Data were extracted by one author (SE), while a second author (EP) read again the full texts of the included trials and independently from the first one checked the data extracted. Discrepancies were identified and resolved after consulting a third author (CK). Any data that were not described in the article were calculated from existing data, if possible, or were tried to be obtained by contacting the authors.

### Risk of bias

The risk of bias of the included non-randomized studies was assessed with the Cochrane Collaboration’s ROBINS-I tool (“Risk Of Bias In Non-randomized Studies of Interventions”) [20]. Assessment of the risk of bias within individual trials was likewise independently performed by two authors (SE, EP) and discrepancies were resolved by consulting a third author (CK).

### Data synthesis and summary measures

Available data were summarized and considered suitable for pooling. Where data were missing, they were calculated by the authors to make the effort to include all existing trials in the analysis. In multi-arm trials with multiple similar intervention groups compared to a control group, similar trial arms were first pooled and then compared to the control group.

To include different measuring methods of relapse, relative relapse extent in % as main outcome variable was used. To calculate this percentage, the absolute differences of the metric and angular cephalometric measurements between the time immediately before (Tpre) and after the surgical intervention (T0) as well as the absolute differences between the time immediately after the surgical intervention (T0) and a period of following-up (T1 at least 1 year post-treatment, T2 not mandatory, but longer than T1) were firstly calculated. After that the relapse extent in % was derived by forming the ratio as |T0–T1|/|T0-Tpre| × 100% or |T0–T2|/|T0-Tpre| × 100%. Then the relapse extent was categorized according to the scheme of Bailey et al. [2] as follows: highly stable in case of ≤ 10% of relapse; stable in case of < 20% of relapse and problematic in case of ≥ 20% of relapse. Frequency distributions of the dependent categorical outcome parameter relative relapse extent and for the predictive independent categorical parameter operative procedure were calculated. The respective measurement parameters of the individual studies were grouped into sagittal or vertical parameters and dental, skeletal or soft tissue parameters (Table 1). The most frequently occurring relapse grade (highly stable, stable, problematic) across all evaluated parameters within each category (skeletal, dental, soft tissue) in each dimension (sagittal, vertical) and in total in the sagittal and vertical dimension was considered as the relative relapse extent of the respective intervention.

**Table 1:** Summary of findings

	I		II		III		IV		Total		Grade
	Unimax mand	Bimax	Unimax mand	Bimax	Unimax	Bimax	Unimax mand	Bimax	Unimax* mand	Bimax**	
*Total*											
Sagittal	++	++	+			T1++/T2−	++		++	++	
Vertical	++	++	−			T1++/T2−	−		++	++	
*Skeletal*											
Sagittal	++	++	+			T1+/T2−	++		++	++/+	
Vertical	++/−	++	−			T1++/T2−	+/−		++/−	++	Low
*Dental*											
Sagittal	++	++	++/+			T1++/T2++	++		++	++	
Vertical	++	++	−			T1++/T2++	−		++/−	++	
*Soft tissue*											
Sagittal	+/−	++								++	
Vertical	++	++				T1+/T2−				++/+	

++ Highly stable

+ Stable

− Problematic

I—study of Ana de Lourdes Sá de Lir et al.: Long-term skeletal and profile stability after surgica-orthodontic treatment of Class II and Class III malocclusion

II—study of P R H Venkategowda et al.: Stability of Vertical, Horizontal and Angular Parameters Following Superior Repositioning of Maxilla by Le Fort I Osteotomy: A cephalometric study

III—study of J A Miguel et al.: Long-term stability of two-jaw surgery for treatment of mandibular deficiency and vertical maxillary excess

IV—study of J Paunonen et al.: Long-term stability of mandibular advancement with bilateral sagittal split osteotomy

*Evaluation in total only possible for study I group unimax and study IV because of the same intervention

**Evaluation in total only possible for study I group bimax and study III because of the same intervention, evaluation for T1

The table shows the distribution of stability frequencies for every study after categorizing stability according to Bailey et al. The most frequently occurring relapse grade (highly stable, stable, problematic) across all evaluated parameters within each category (skeletal, dental, soft tissue) in each dimension (sagittal, vertical) and in total in the sagittal and vertical dimension was considered as the relative relapse extent of the respective intervention

### Additional analyses

Due to the lack of studies, which fulfilled the eligibility criteria, a meta-analysis could not be performed. Hence, neither heterogeneity nor publication bias could be assessed.

The overall quality of clinical recommendations (confidence in effect estimates) for each of the main outcomes was rated by using the Grades of Recommendation, Assessment, Development and Evaluation approach (GRADE) [6].

## Results

### Study selection

**Fig. 1:**
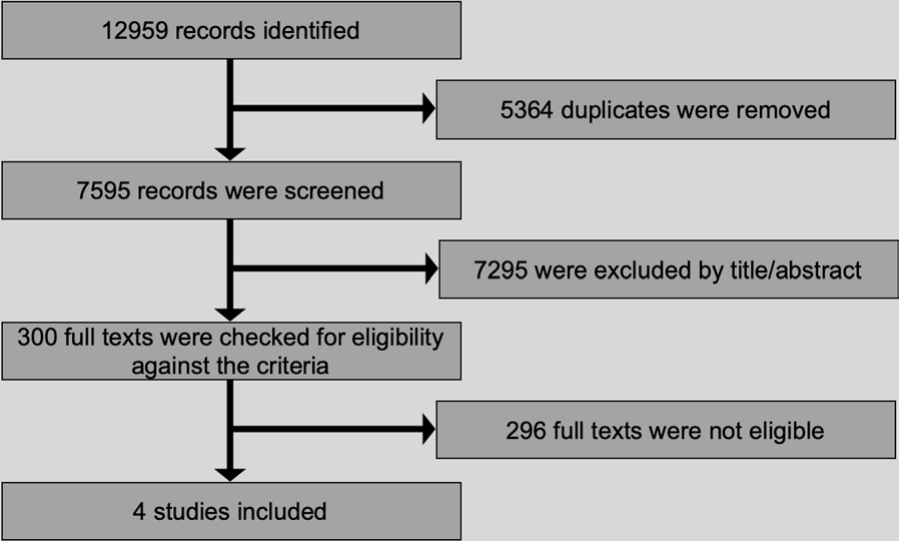
PRISMA flow diagram for the identification and selection of eligible studies

The electronic literature search yielded 12,959 results (Fig. 1). After duplicate removal and screening of titles or abstracts against the predefined eligibility criteria (Additional file 1: Appendix 3) the full texts of 300 papers were checked. Finally, 4 papers pertaining to 4 unique studies (4 retrospective non-randomized studies), which were published as journal papers, were included [12, 15, 19, 21].

### Study characteristics

The studies were conducted in university hospitals (n = 4) and in one study additionally in private practices and originated from four different countries: Brasil, India, USA, Finland [12, 15, 19, 21]. A total of 132 (range 10 to 50) patients were included, which does not exactly correspond to the total number of participants in the primary studies, because only Angle Class II patients were taken into consideration for this review. Out of 3 studies reporting on patients´ gender, there were at least 86 female patients and at least 36 male patients. The range of the patients´ age was 14–63 years.

Two of the included studies explicitly assessed single jaw interventions [15, 19] and two explicitly bimaxillary interventions [12, 19], whereas one of them did not differentiate patients with maxillary intervention only and patients with a bimaxillary procedure [21]. The evaluated interventions consisted of mandibular advancement surgery by bilateral sagittal split osteotomy, maxillary impaction by LeFort I osteotomy and bimaxillary procedures, which combined both. All included studies reported about perioperative orthodontic treatment, which involved a preadjusted Edgewise appliance and premolar extractions [21], straight-wire orthodontic technique [15], cointerventions like genioplasty [12, 15] and unspecified perioperative orthodontic treatment.

The assessed post-operative follow-up period covered a range of at least one year up to maximum 8 years. Evaluation of stability was based on common linear and angular measurements on cephalometric radiographs in all included studies.

### Risk of bias

**Fig. 2:**
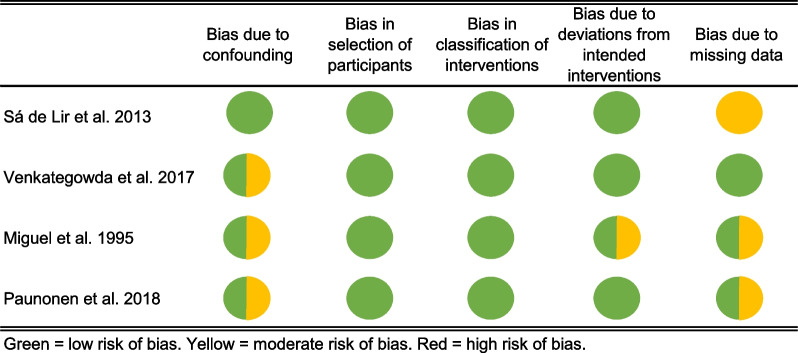
Risk of bias assessment

All four included studies (Fig. 2) were non-randomized cohort studies, which presented some issues that increased their risk for bias, but the overall risk of bias was found to be low in two studies [19, 21] and moderate for the other two [12, 15].

All studies were preconceived to be in moderate risk of baseline confounding and identifying confounding, because even though preoperative variables were defined, the decision criteria for a procedure and the kind of procedure was not predefined in any study. In two studies the patients did not get all the same cointerventions or it was not reported transparently, which patient received which cointervention, therefore they were judged as moderate risk of bias due to deviation from the intended interventions [12, 21]. All studies did not provide the measurement results of the individual patients and were judged to be of moderate risk of bias due to missing data.

Finally, all studies were judged to be at low risk of bias for classification of interventions, selection of participants, measurement of the outcome and in selection of reported results.

### Data synthesis

A total of 4 studies with 132 patients with Angle class II dysgnathia were eligible for data synthesis. There were not enough data to perform a meta-analysis. The studies presented too little equal reference points for evaluation of relapse after surgery. Therefore, a comparison of relapse between the different procedures was not feasible. To answer the question, if the hierarchy of stability of Proffit et al. [17, 18] is supported by currently available evidence, frequencies of the categories of Bailey et al. [2] were evaluated for the respective dimensions (sagittal, vertical and dental, skeletal and soft tissue) for each study (Table 1). Only three studies were comparable because of the same interventions. The highly stable results in vertical and sagittal dimensions of single-jaw procedures at the mandible [15, 19] complied with Proffit’s et al. [17, 18] hierarchy, but our findings indicated contrary results for skeletal vertical stability and dental vertical stability. Bimaxillary procedures [12, 19] (mandibular advancement with superior reposition of the maxilla) to correct Angle class II situations exceeded the hierarchy because of highly stable results.

### Results of individual studies

Across single studies, some discrepancies arose. While Sà de Lir et al. [19] demonstrated the expected outcome for the single-jaw group and only indicated contrary results for skeletal vertical and soft tissue sagittal stability, Paunonen et al. [15] corresponded with the “hierarchy of stability” only regarding sagittal stability (Table 1). The expectation of highly stable results after superior repositioning of the maxilla was not confirmed by Venkategowda et al. [21]. Whereas the bimaxillary group of Sà de Lir et al. [19] exceeded the hierarchy with highly stable results, Miguel et al. [12] could not confirm this with problematic skeletal stability after a middle-term follow up (at least 5 years post-operative).

### Additional analyses

Due to limited data, subgroup analysis and meta-analysis could not be performed. The quality of evidence (Table 1) for the main outcome of stability after surgical correction of Angle Class II dysgnathia was low, due to the inclusion of non-randomized studies. Besides that, some inconsistency in the results appeared and only small populations were assessed.

### Sensitivity analysis

No sensitivity analysis could be performed by rendering non-randomized studies, as only non-randomized studies were included.

## Discussion

The present systematic review summarizes evidence from non-randomized studies on relapse after class II orthognathic surgery. Four studies were finally included according to the review’s eligibility criteria. The data of following-up 132 patients after their surgical interventions indicated that bimaxillary surgery and mandibular advancement to correct Angle class II dysgnathia are highly stable interventions as determined by less than 10% of significant posttreatment change [2]. Only one study was found to evaluate the surgical correction by superior repositioning of the maxilla [21]. The results were highly stable for sagittal dental parameters and stable for sagittal skeletal parameters. These outcomes correspond partly to the postulated hierarchy of stability of Proffit et al. [17], which only reaches a different conclusion for the stability of bimaxillary surgery. A possible explanation is the small population evaluated, which could overestimate the effects of the procedures [6, 14]. On the other hand, some discrepancies between the results within the studies were demonstrated. The frequencies of skeletal stability in the vertical and mandibular advancement procedures were ambivalent, since highly stable results (++) and problematic results (−) were at the same level. The evaluation in total of single jaw interventions within the mandible yielded identical findings, also in the dental vertical dimension. Potential reasons for these mismatches are that matching variables across retrospective cephalometric studies, such as reference points, the surgical technique (unisegmental vs. segmental splits), the degree of jaw movement, and the nature of fixation (rigid vs. non-rigid) makes a direct comparison of values difficult [1]. Furthermore, there is a risk of bias expected due to potential deviations from intended interventions. The same risk of bias appears due to missing data in studies.

The studies presented different periods of follow-up, which also can be assumed to impact on results. Surgical healing is assumed to be complete at one year posttreatment. Changes beyond 1 year represent some combination of postural change, compensatory bone remodeling, dental changes and late growth in the pattern that produced the original dentofacial deformity. These changes are probably minimal related to the type of surgery, the type of surgical fixation, and other influences on stability that affect changes during the first year [16]. This can explain the contradictory findings between T1 (one year post-operative) and T2 (at least 5 years post-operative) in the study of Miguel et al. [12]. Another incongruity is the wide age span of the study population (14–63 years). The fact, that patients under 18 have growth potential and conceivably more reactive tissue, biases the oberserved stability after the surgical procedure [3, 4, 8]. Non-transparently reported or different kinds of perioperative treatment regime, such as Edgewise appliance, tooth extractions, additional interventions such as genioplasty, furthermore make comparisons between studies difficult.

Consequently, all these inconsistencies do not lead to a complete coherent result and do not enable a representative statement about the stability after orthognathic surgery to correct class II dysgnathia.

Considering newest evidence like the study of Gaitan-Romero et al. of 2021 [5] aformentioned aspects get reflected. Direct comparisons between studies are rarely possible due to variations in intervention strategies between inherently few studies. Some studies describe directions of the repositioning of the jaws without reference of the kind of intervention. As this review examined relapse rates on the basis of sagittal and vertical dimensions independent of specific landmarks, Gaitan-Romero et al. [5] assessed relapse on the basis of selected landmarks for different dimensions. According to Bailey et al. [2] it is misleading to describe stability in terms of percentage of treatment change that was retained at some follow-up time. So the strenght of this systematic review was to group stability data like Bailey et al. to not overestimate few but outstanding results.

However, next to limitations of this review regarding variations in study design and assessment methodology, meta-analysis was not possible due to small study sizes and heterogenity of data. Further limitations regarded the low quality of evidence due to unexeptional retrospective studies with small sample sizes. An additional limitation of this review was that no study used 3D-Imaging to evaluate relapse and no long term follow-up evaluation was possible.

This research gap requires randomized controlled clinical studies with consistent populations of a representative size to compare the various single jaw and bimaxillary interventions with and among each other and to detect, which variables affect the stability after surgery to what extent.

## Conclusion

Based on available evidence from retrospective non-randomized studies assessing relapse in class II orthognathic surgery, no fundamental deviation from the postulated “hierarchy of stability” from Proffit et al. [17, 18] was found. However, existing evidence on the topic is limited and based mainly on potentially flawed study designs. Future studies, especially randomized studies with transparent reporting of treatment regime, comparable population groups and adequate handling of confounders are needed to address this topic.

## Supplementary Information

**Additional file:** Appendix 1-3.

## Data Availability

All data generated or analysed during this study are included in this published article and its supplementary information file.
