# Supplementary material for: Relapse in class II orthognathic surgery: a systematic review
Source: BMC Oral Health. 2022 Dec 15;22:605. doi: 10.1186/s12903-022-02636-x (PMC9753235; doi:10.1186/s12903-022-02636-x)
Supplement: Supplementary file 1 — Additional file: Appendix 1-3. [file 12903_2022_2636_MOESM1_ESM.pdf]

Appendix 1 - PRISMA 2020 Checklist

Section and Topic	Item #	Checklist item	Location where item is reported
**TITLE**			
Title	1	Identify the report as a systematic review.	1
**ABSTRACT**			
Abstract	2	See the PRISMA 2020 for Abstracts checklist.	1-2
**INTRODUCTION**			
Rationale	3	Describe the rationale for the review in the context of existing knowledge.	2-3
Objectives	4	Provide an explicit statement of the objective(s) or question(s) the review addresses.	2-3
**METHODS**			
Eligibility criteria	5	Specify the inclusion and exclusion criteria for the review and how studies were grouped for the syntheses.	4-5
Information sources	6	Specify all databases, registers, websites, organisations, reference lists and other sources searched or consulted to identify studies. Specify the date when each source was last searched or consulted.	5
Search strategy	7	Present the full search strategies for all databases, registers and websites, including any filters and limits used.	5-6<br>Appendix 2
Selection process	8	Specify the methods used to decide whether a study met the inclusion criteria of the review, including how many reviewers screened each record and each report retrieved, whether they worked independently, and if applicable, details of automation tools used in the process.	5-6
Data collection process	9	Specify the methods used to collect data from reports, including how many reviewers collected data from each report, whether they worked independently, any processes for obtaining or confirming data from study investigators, and if applicable, details of automation tools used in the process.	6
Data items	10a	List and define all outcomes for which data were sought. Specify whether all results that were compatible with each outcome domain in each study were sought (e.g. for all measures, time points, analyses), and if not, the methods used to decide which results to collect.	7
	10b	List and define all other variables for which data were sought (e.g. participant and intervention characteristics, funding sources). Describe any assumptions made about any missing or unclear information.	4,7
Study risk of bias assessment	11	Specify the methods used to assess risk of bias in the included studies, including details of the tool(s) used, how many reviewers assessed each study and whether they worked independently, and if applicable, details of automation tools used in the process.	6
Effect measures	12	Specify for each outcome the effect measure(s) (e.g. risk ratio, mean difference) used in the synthesis or presentation of results.	7
Synthesis methods	13a	Describe the processes used to decide which studies were eligible for each synthesis (e.g. tabulating the study intervention characteristics and comparing against the planned groups for each synthesis (item #5)).	6-7
	13b	Describe any methods required to prepare the data for presentation or synthesis, such as handling of missing summary statistics, or data conversions.	6-7
	13c	Describe any methods used to tabulate or visually display results of individual studies and syntheses.	6-7; 11
	13d	Describe any methods used to synthesize results and provide a rationale for the choice(s). If meta-analysis was performed, describe the model(s), method(s) to identify the presence and extent of statistical heterogeneity, and software package(s) used.	6-8;11
	13e	Describe any methods used to explore possible causes of heterogeneity among study results (e.g. subgroup analysis, meta-regression).	6-8
	13f	Describe any sensitivity analyses conducted to assess robustness of the synthesized results.	6-8
Reporting bias assessment	14	Describe any methods used to assess risk of bias due to missing results in a synthesis (arising from reporting biases).	6

Appendix 1 - PRISMA 2020 Checklist

Section and Topic	Item #	Checklist item	Location where item is reported
Certainty assessment	15	Describe any methods used to assess certainty (or confidence) in the body of evidence for an outcome.	8
**RESULTS**			
Study selection	16a	Describe the results of the search and selection process, from the number of records identified in the search to the number of studies included in the review, ideally using a flow diagram.	8
	16b	Cite studies that might appear to meet the inclusion criteria, but which were excluded, and explain why they were excluded.	9; Appendix 3
Study characteristics	17	Cite each included study and present its characteristics.	9
Risk of bias in studies	18	Present assessments of risk of bias for each included study.	10
Results of individual studies	19	For all outcomes, present, for each study: (a) summary statistics for each group (where appropriate) and (b) an effect estimate and its precision (e.g. confidence/credible interval), ideally using structured tables or plots.	10-12
Results of syntheses	20a	For each synthesis, briefly summarise the characteristics and risk of bias among contributing studies.	10-11
	20b	Present results of all statistical syntheses conducted. If meta-analysis was done, present for each the summary estimate and its precision (e.g. confidence/credible interval) and measures of statistical heterogeneity. If comparing groups, describe the direction of the effect.	10-11
	20c	Present results of all investigations of possible causes of heterogeneity among study results.	12
	20d	Present results of all sensitivity analyses conducted to assess the robustness of the synthesized results.	12
Reporting biases	21	Present assessments of risk of bias due to missing results (arising from reporting biases) for each synthesis assessed.	10
Certainty of evidence	22	Present assessments of certainty (or confidence) in the body of evidence for each outcome assessed.	12
**DISCUSSION**			
Discussion	23a	Provide a general interpretation of the results in the context of other evidence.	12-14
	23b	Discuss any limitations of the evidence included in the review.	12-14
	23c	Discuss any limitations of the review processes used.	12-14
	23d	Discuss implications of the results for practice, policy, and future research.	14
**OTHER INFORMATION**			
Registration and protocol	24a	Provide registration information for the review, including register name and registration number, or state that the review was not registered.	4;15
	24b	Indicate where the review protocol can be accessed, or state that a protocol was not prepared.	4;15
	24c	Describe and explain any amendments to information provided at registration or in the protocol.	4;15
Support	25	Describe sources of financial or non-financial support for the review, and the role of the funders or sponsors in the review.	14
Competing interests	26	Declare any competing interests of review authors.	15
Availability of data, code and other materials	27	Report which of the following are publicly available and where they can be found: template data collection forms; data extracted from included studies; data used for all analyses; analytic code; any other materials used in the review.	15

Appendix 2 – search history

Performance of research and documentation
Dr. Helge Knüttel, librarian of university of Regensburg

Protocol

Limitations

- Date of publication: no limits
- Language: no limits

Sources for research

Databases

Name of Database	Provider / Interface	Time period covered
EMBASE	Ovid	1974–current
MEDLINE	Ovid	1946–current
Cochrane Library:		
CDRS (Cochrane Database of Systematic Reviews)	Wiley Online Library	1995–current
CENTRAL (Cochrane Central Register of Controlled Trials)	Wiley Online Library	1948–current
DARE (Database of Abstracts of Reviews of Effect)	Wiley Online Library	1995–2015
NHS Economic Evaluation Database	Wiley Online Library	1994– 2015
HTA (Health Technology Assessment Database)	Wiley Online Library	1988– 2015
Science Citation Index Expanded	Web of Science	1965–current
ClinicalTrials.gov	NLM	---
International Clinical Trials Registry Platform	WHO	---
Google Scholar	Google	---

Prepared searchterm
This searchterm was prepared and has a form which could be refined easily then the exported searchterm of the search platform
Searchterm
Entered terms may changed by the search platform. The exported version of the platform is documented

EMBASE

Datenbank	
Embase 1974 to 2019 July 15 (oemezd)	
Platform (potentially incl. Version)	
Ovid	
Time period of platform	
1974–2019	
Time limitation	
none	
Standard-searchfilter	
none	
other limitations	
none	
Date of search	
2019-07-16	
Executed by	
Helge Knüttel	
Peer Review	
no	
Quantity of matches (with doublets)	
4548	
Exportformat of matches	
OvidSP für Citavi (Citavi-Importfilter: OvidSP)	
4548 matches were totally exported in portions of the maximum of 1000<br>$ for file in \`find . -name 'kaluza_2738_2019-07-16_EMBASE_r*-*.ovd' -print\` ; do echo $file; grep "^DB - Embase" $file \| wc -l ; done<br>./kaluza_2738_2019-07-16_EMBASE_r0001-1000.ovd<br>1000<br>./kaluza_2738_2019-07-16_EMBASE_r1001-2000.ovd<br>1000<br>./kaluza_2738_2019-07-16_EMBASE_r2001-3000.ovd<br>1000<br>./kaluza_2738_2019-07-16_EMBASE_r3001-4000.ovd<br>1000<br>./kaluza_2738_2019-07-16_EMBASE_r4001-4548.ovd<br>548<br><br>export-file within doublets : (Export-)Felds UI (= Accession Number/AN) enthalten:<br>$ for file in \`find . -name 'kaluza_2738_2019-07-16_EMBASE_r*-*.ovd' -print\` ; do echo $file; grep "^UI - " $file | sort | uniq | wc -l ; done<br>./kaluza_2738_2019-07-16_EMBASE_r0001-1000.ovd<br>995<br>./kaluza_2738_2019-07-16_EMBASE_r1001-2000.ovd<br>1000	

./kaluza_2738_2019-07-16_EMBASE_r2001-3000.ovd
1000
./kaluza_2738_2019-07-16_EMBASE_r3001-4000.ovd
1000
./kaluza_2738_2019-07-16_EMBASE_r4001-4548.ovd
548

Consindering the total quantity of matches of 4548 database entries within all files, there are 4538 individual matches included (ANs):
$ grep --no-filename "^UI - " kaluza_2738_2019-07-16_EM
BASE_r*-*.ovd | sort | uniq | wc -l
4538

The different export files were pooled to ease the import an reduce error source
$ cat kaluza_2738_2019-07-16_EMBASE_r*-*.ovd >> kaluza_2
738_2019-07-16_EMBASE_allrecords.ovd

Search history

#	Search statement	Record count	Annotation
1	Retrognathia/ or micrognathia/ or (("class II" or "class 2") and malocclusion$).ti,ab,kw. or (posterior adj3 occlusion$).ti,ab,kw. or (distoclusion$ or disto-occlusion$ or distocclusion$).ti,ab,kw. or (distal adj (occlusion$ or bite$)).ti,ab,kw. or retrognath$.ti,ab,kw. or (prominent adj3 upper adj3 teeth).ti,ab,kw. or (overjet$ or over jet$).ti,ab,kw. or (overbite$ or over bite$).ti,ab,kw. or (deepbite$ or deep bite$).ti,ab,kw. or (mandib$ adj3 (micrognath$ or retropos$ or retrus$)).ti,ab,kw. or (maxill$ adj3 (prognath$ or anteposition$)).ti,ab,kw.	14268	angle class II malocclusion
2	exp orthognathic surgery/ or oral surgery/ or orthognath*.ti,ab,kw. or (orthodont* and (surgery or surgeries or surgical)).ti,ab,kw. or ((mandib* or jaw* or maxill* or bimaxillary or Prognath* or Retrognath* or occlusion or malocclusion or angle or "class II" or "class 2" or nonocclusion or overbite* or "over bite*" or openbite* or "open bite*" or "deep bite*" or deepbite* or "over jet*" or overjet* or dentofacial or "dento facial" or ramal or ramus) adj3 (surg* or procedure* or operation* or operative or correct* or osteotom* or advancement or reduction* or reposition* or setback or advancement or retropos* or retrus* or fixation* or distract* or resect*)).ti,ab,kw. or (mandibulotom* or mandibulectom* or hemimandibulectom* or maxillotom* or maxillectom*).ti,ab,kw. or ("Le Fort" or lefort).ti,ab,kw. or ((exp jaw malformation/ or malocclusion/) and su.fs.)	77357	orthognatic surgery
3	1 and 2	4548	angle class II malocclusion AND orthognatic surgery

MEDLINE

Database	
Ovid MEDLINE(R) ALL 1946 to July 15, 2019 (medall)	
Platform (potentially incl. Version)	
Ovid	
Time period of database	
about 1946–2019	
Time limitation	
none	
Standard-searchfilter	
none	
Other limitations	
Removal of animal-only studies via check tags.	
Date of search	
2019-07-16	
Executed by	
Helge Knüttel	
Peer Review	
no	
Quantity of matches with doublets	
5284	
Exportformat of matches	
OvidSP für Citavi (Citavi-Importfilter: OvidSP)	
5284 matches were exported totally in portions oft he maximum of 1000 :<br>$ for file in \`find . -name 'kaluza_2738_2019-07-16_MEDLINE_r*-*.ovd' -print\` ; do echo $file; grep "^DB - Ovid MEDLINE(R)" $file \| wc -l ; done<br>./kaluza_2738_2019-07-16_MEDLINE_r0001-1000.ovd<br>1000<br>./kaluza_2738_2019-07-16_MEDLINE_r1001-2000.ovd<br>1000<br>./kaluza_2738_2019-07-16_MEDLINE_r2001-3000.ovd<br>1000<br>./kaluza_2738_2019-07-16_MEDLINE_r3001-4000.ovd<br>1000<br>./kaluza_2738_2019-07-16_MEDLINE_r4001-5000.ovd<br>1000<br>./kaluza_2738_2019-07-16_MEDLINE_r5001-5284.ovd<br>284<br><br>In on Export-file are doublets: (Export-)Felds UI (= Accession Number/AN) enthalten:<br>$ for file in \`find . -name 'kaluza_2738_2019-07-16_MEDLINE_r*-*.ovd' -print\` ; do echo $file; grep "^UI - " $file | sort | uniq | wc -l ; done<br>./kaluza_2738_2019-07-16_MEDLINE_r0001-1000.ovd<br>997	

```
./kaluza_2738_2019-07-16_MEDLINE_r1001-2000.ovd
1000
./kaluza_2738_2019-07-16_MEDLINE_r2001-3000.ovd
1000
./kaluza_2738_2019-07-16_MEDLINE_r3001-4000.ovd
1000
./kaluza_2738_2019-07-16_MEDLINE_r4001-5000.ovd
1000
./kaluza_2738_2019-07-16_MEDLINE_r5001-5284.ovd
284
```

Consindering the total quantity of matches of 5284 database entries within all files, there are 5281 individual matches included (ANs):

```
$ grep --no-filename "^UI  - " kaluza_2738_2019-07-16_ME
DLINE_r*-*.ovd | sort | uniq | wc -l
5281
```

Search history

#	Search statement	Record count	Annotation
1	exp Malocclusion, Angle Class II/ or Retrognathia/ or Micrognathism/ or (("class II" or "class 2") and malocclusion$).ti,ab,kf. or (posterior adj3 occlusion$).ti,ab,kf. or (distoclusion$ or disto-occlusion$ or distocclusion$).ti,ab,kf. or (distal adj (occlusion$ or bite$)).ti,ab,kf. or retrognath$.ti,ab,kf. or (prominent adj3 upper adj3 teeth).ti,ab,kf. or (overjet$ or over jet$).ti,ab,kf. or (overbite$ or over bite$).ti,ab,kf. or (deepbite$ or deep bite$).ti,ab,kf. or (mandib$ adj3 (micrognath$ or retropos$ or retrus$)).ti,ab,kf. or (maxill$ adj3 (prognath$ or anteposition$)).ti,ab,kf.	14012	angle class II malocclusion
2	exp orthognathic surgical procedures/ or osteotomy, le fort/ or osteotomy, sagittal split ramus/ or Osteotomy/ or Surgery, Oral/ or Oral Surgical Procedures/ or Jaw Fixation Techniques/ or Mandibular Advancement/ or exp Orthognathic Surgical Procedures/ or ((Jaw Abnormalities/ or exp Malocclusion/ or Micrognathism/ or Retrognathia/ or exp Jaw/) and su.fs.) or orthognath*.ti,ab,kf. or (orthodont* and (surgery or surgeries or surgical)).ti,ab,kf. or ((mandib* or jaw* or maxill* or bimaxillary or Prognath* or Retrognath* or occlusion or malocclusion or angle or "class II" or "class 2" or nonocclusion or overbite* or "over bite*" or openbite* or "open bite*" or "deep bite*" or deepbite* or "over jet*" or overjet* or dentofacial or "dento facial" or ramal or ramus) adj3 (surg* or procedure* or operation* or operative or correct* or osteotom* or advancement or reduction* or reposition* or setback or advancement or retropos* or retrus* or fixation* or distract* or resect*)).ti,ab,kf. or (mandibulotom* or mandibulectom* or hemimandibulectom* or maxillotom* or maxillectom*).ti,ab,kf. or ("Le Fort" or lefort).ti,ab,kf. or ((jaw abnormalities/ or micrognathism/ or retrognathia/ or malocclusion/) and su.fs.)	102499	orthognatic surgery
3	1 and 2	5328	angle class II malocclusion AND orthognatic surgery
4	3 not (animals/ not human/)	5284	exclusion of animal-only studies

Cochrane Library

Database	
Cochrane Library:<br>• CDRS (Cochrane Database of Systematic Reviews)<br>• DARE (Database of Abstracts of Reviews of Effect)<br>• CENTRAL (Cochrane Central Register of Controlled Trials)<br>• HTA (Health Technology Assessment Database)<br>• EED (NHS Economic Evaluation Database)	
Platform (potentially incl. Version)	
Wiley Online Library	
Time period of database	
• CDRS (Cochrane Database of Systematic Reviews): 1993–current<br>• DARE (Database of Abstracts of Reviews of Effect): 1995–2015<br>• CENTRAL (Cochrane Central Register of Controlled Trials): 1948–current<br>• HTA (Health Technology Assessment Database): 1994– 2017<br>• EED (NHS Economic Evaluation Database): 1988– 2015	
Time limitation	
none	
Standard-searchfilter	
none	
Other limitations	
none	
Date of search	
2019-07-16	
Executed by	
Helge Knüttel	
Peer Review	
no	
Quantity of matches with doublets	
total: 353<br>• Cochrane Reviews: 5<br>• Trials: 348	
Exportformat of matches	
RIS (Reference Manager)	
The matches were exported in RIS-files in total. The search interface allows this only separated for every individual database of Cochrane Library:<br><br>$ for file in \`find . -name 'kaluza_2738_2019-07-16_Cochrane*.ris' -print\` ; do echo $file; grep "^TY - " $file | wc -l ; done<br>./kaluza_2738_2019-07-16_Cochrane_CDSR.ris<br>5<br>./kaluza_2738_2019-07-16_Cochrane_CENTRAL.ris<br>348	

Search history

ID	Search	Hits
#1	(((class NEXT II) OR (class NEXT 2)) and malocclusion*):ti,ab,kw	477
#2	(posterior NEAR/3 occlusion*):ti,ab,kw	26
#3	(distoclusion* OR disto-occlusion* OR distocclusion*):ti,ab,kw	9
#4	(distal NEXT (occlusion* OR bite*)):ti,ab,kw	32
#5	(retrognath*):ti,ab,kw	83
#6	(prominent NEAR/3 upper NEAR/3 teeth):ti,ab,kw	3
#7	(overjet* OR (over NEXT jet*)):ti,ab,kw	220
#8	(overbite* OR (over NEXT bite*)):ti,ab,kw	162
#9	(deepbite* OR (deep NEXT bite*)):ti,ab,kw	21
#10	(mandib* NEAR/3 (micrognath* OR retropos* OR retrus*)):ti,ab,kw	32
#11	(maxill* NEAR/3 (prognath* OR anteposition*)):ti,ab,kw	5
#12	#1 OR #2 OR #3 OR #4 OR #5 OR #6 OR #7 OR #8 OR #9 OR #10 OR #11	749
#13	(orthognath*):ti,ab,kw	445
#14	(orthodont* AND (surgery OR surgeries OR surgical)):ti,ab,kw	465
#15	((mandib* OR jaw* OR maxill* OR bimaxillary OR Prognath* OR Retrognath* OR occlusion OR malocclusion OR angle OR (class NEXT II) OR (class NEXT 2) OR nonocclusion OR overbite* OR (over NEXT bite*) OR openbite* OR (open NEXT bite*) OR (deep NEXT bite*) OR deepbite* OR (over NEXT jet*) OR overjet* OR dentofacial OR (dento NEXT facial) OR ramal OR ramus) NEAR/3 (surg* OR procedure* OR operation* OR operative OR correct* OR osteotom* OR advancement OR reduction* OR reposition* OR setback OR advancement OR retropos* OR retrus* OR fixation* OR distract* OR resect*)):ti,ab,kw	5013
#16	(mandibulotom* OR mandibulectom* OR hemimandibulectom* OR maxillotom* OR maxillectom*):ti,ab,kw	47
#17	((Le NEXT Fort) OR lefort):ti,ab,kw	194
#18	#13 OR #14 OR #15 OR #16 OR #17	5503
#19	#12 AND #18	353

Science Citation Index Expanded / Web of Science

Database	
Science Citation Index Expanded (SCI-EXPANDED)<br>Data last updated: 2019-07-15	
Plattform (potentially incl. Version)	
Web of Science	
Time period of database	
Science Citation Index Expanded (SCI-EXPANDED) --1965-present	
Time limitation	
none	
Standard-searchterm	
none	
Other limitations	
none	
Date of search	
2019-07-16	
Executed by	
Helge Knüttel	
Peer Review	
no	
Quantity of matches with doublets	
2024	
Exportformat of matches	
Other reference software<br>Import in Citavi mit dem Textfilter „Web of Scienc (WoS)“	
Export oft he search history was not possible<br><br>Quantity of exported matches was verified:<br>> grep "^ER$" kaluza_2738_2019-07-16_WoS_r*-*.txt | wc -l<br>> 2024<br><br>Consindering the total quantity of matches of 2024 database entries within all files, there are 2024 individual matches included (ANs):<br>> grep --no-filename "^UT " kaluza_2738_2019-07-16_WoS_r*-*.txt | sort | uniq | wc –l<br>2024<br><br>The different exoport files were pooled to ease the import and reduce error sources<br>unite_wos_files.bash kaluza_2738_2019-07-16_WoS_r*.txt > kaluza_2738_2019-07-16_WoS_allrecords.txt	

Searchhistory

Set	Results	Save History / Create Alert Open Saved History	Edit Sets	Combine Sets ○ AND ○ OR Combine	Delete Sets Select All ✕ Delete
# 7	2,024	#6 OR #5 OR #4 OR #3<br>*Indexes=SCI-EXPANDED Timespan=All years*	Edit	☐	☐
# 6	129	**CITED AUTHOR:** (Proffit W*) *AND* **CITED WORK:** (HEAD FACE MEDICINE)<br>*Indexes=SCI-EXPANDED Timespan=All years*		☐	☐
# 5	64	**CITED AUTHOR:** (Bailey L*) *AND* **CITED WORK:** ("AMERICAN JOURNAL OF ORTHODONTICS AND DENTOFACIAL ORTHOPEDICS" OR "AMERICAN JOURNAL OF ORTHODONTICS AND DENTOFACIAL ORTHOPEDICS OFFICIAL PUBLICATION OF THE AMERICAN ASSOCIATION OF ORTHODONTISTS ITS CONSTITUENT SOCIETIES AND THE AMERICAN BOARD OF ORTHODONTICS") *AND* **CITED YEAR:** (2004)<br>*Indexes=SCI-EXPANDED Timespan=All years*		☐	☐
# 4	171	**CITED AUTHOR:** (Proffit W*) *AND* **CITED WORK:** (Int J Adult Orthodon Orthognath Surg) *AND* **CITED YEAR:** (1996) *AND* **CITED VOLUME:** (11) *AND* **CITED PAGES:** (191)<br>*Indexes=SCI-EXPANDED Timespan=All years*		☐	☐
# 3	1,782	#1 AND #2<br>*Indexes=SCI-EXPANDED Timespan=All years*	Edit	☐	☐
# 2	40,644	(TS=(orthognath*)) OR (TS=(orthodont* AND ("surgery" OR "surgeries" OR "surgical"))) OR (TS= ((mandib* OR jaw* OR maxill* OR "bimaxillary" OR Prognath* OR Retrognath* OR "occlusion" OR "malocclusion" OR "angle" OR "class II" OR "class 2" OR "nonocclusion" OR overbite* OR "over bite*" OR openbite* OR "open bite*" OR "deep bite*" OR deepbite* OR "over jet*" OR overjet* OR "dentofacial" OR "dento facial" OR "ramal" OR "ramus") NEAR/2 (surg* OR procedure* OR operation* OR "operative" OR correct* OR osteotom* OR "advancement" OR reduction* OR reposition* OR "setback" OR "advancement" OR retropos* OR retrus* OR fixation* OR distract* OR resect*))) OR (TS=(mandibulotom* OR mandibulectom* OR hemimandibulectom* OR maxillotom* OR maxillectom*)) OR (TS=("Le Fort" OR "lefort"))<br>*Indexes=SCI-EXPANDED Timespan=All years*	Edit	☐	☐
# 1	5,307	(TS=(("class II" OR "class 2") AND malocclusion*)) OR (TS=("posterior" NEAR/2 occlusion*)) OR (TS=(distoclusion* OR disto-occlusion* OR distocclusion*)) OR (TS=("distal" NEAR/0 (occlusion* OR bite*))) OR (TS=(retrognath*)) OR (TS=("prominent" NEAR/2 "upper" NEAR/2 "teeth")) OR (TS=(overjet* OR "over jet*")) OR (TS=(overbite* OR "over bite*")) OR (TS=(deepbite* OR "deep bite*")) OR (TS=(mandib* NEAR/2 (micrognath* OR retropos* OR retrus*))) OR (TS=(maxill* NEAR/2 (prognath* OR anteposition*)))<br>*Indexes=SCI-EXPANDED Timespan=All years*	Edit	☐	☐
				○ AND ○ OR Combine	Select All ✕ Delete

Google Scholar

Database	
Google Scholar	
Plattform (potentially incl. Version)	
Search with programm “Harzing's Publish or Perish”, Version 6.49.6406.7079	
Time period of database	
?-present	
Time limitation	
none	
Standard-searchterm	
none	
Other limitations	
none	
Date of search	
2019-07-16	
Executed by	
Helge Knüttel	
Peer Review	
no	
Quantity of mathces with doublets	
200 (only first 200 exported)	
Exportformat of matches	
RIS, Endnote	
Searchstring typed in the field „Any of the words“	

Searchhistory

Searchstring for Google Scholar generated with 2dsearch (https://app.2dsearch.com/). Illustration:

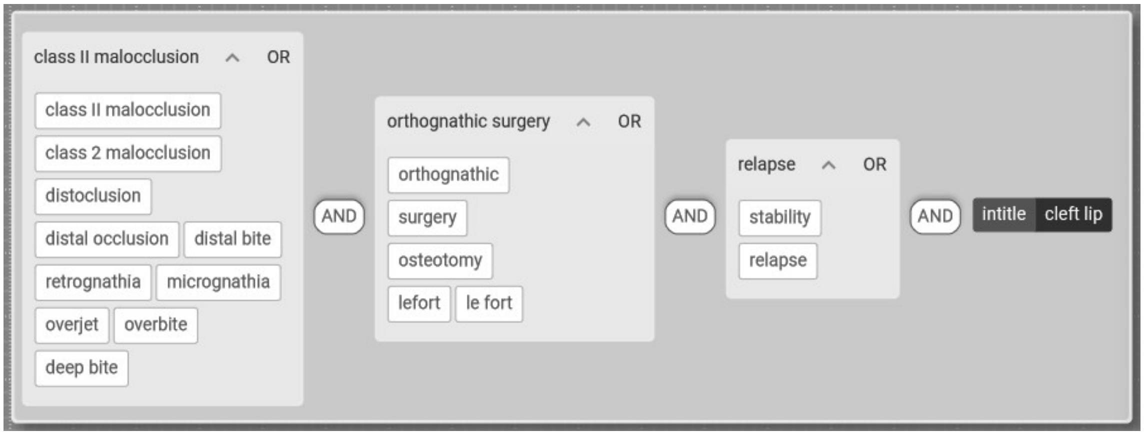

Publish or Perish 6.49.6406.7079
Search terms
Any of the words: ("class II malocclusion"|"class 2 malocclusion"|distoclusion|"distal occlusion"|"distal bite"|retrognathia|micrognathia|overjet|overbite|"deep bite")(orthognathic|surgery|osteotomy|lefort|"le fort")(stability|relapse) -intitle:"cleft lip"
Years: all

WHO's International Clinical Trials Registry Platform

Database	
WHO's International Clinical Trials Registry Platform	
Plattform (potentially incl. Version)	
http://apps.who.int/trialsearch/<br>Version 3.6	
Time period of database	
?–current	
Time limitation	
none	
Standard-searfilter	
none	
Other limitations	
none	
Date of search	
2019-07-16	
Executed by	
Helge Knüttel	
Peer Review	
no	
Quantity of matches with doublets	
354 records for 312 trials	
Exportformat of matches	
XML, CSV	
Option „Without Synonyms“ was performed because oft the potential risk of too many irrelevant matches. Phrase search did not work. There were often matches without the searched phrase e.g.: over jet.<br><br>Quantity of matches verified in XML-file (file used for further process)<br>grep "^ <Trial>" kaluza_2738_2019-07-16_ICTRP_results.xml | wc -l<br>312<br><br>Quantity of matches verified in CSV-file:<br>wc -l kaluza_2738_2019-07-16_ICTRP_results.csv<br>316<br><br>Verification showed individual line breaks wihtin the fields. That’s why quantity of lines is higher than quantity of records	

Searchhistory

354 records for 312 trials found for: malocclusion OR posterior occlusion OR class II AND occlusion OR class 2 AND occlusion OR distoclusion OR disto-occlusion OR distocclusion OR distal occlusion OR distal bite OR retrognath* OR prominent upper teeth OR overjet OR over-jet OR overbite over-bite OR deepbite OR deep-bite OR mandib* AND micrognath* OR mandib* AND retropos* OR mandib* AND retrus* OR maxill* prognath OR maxill* AND antepos*

ClinicalTrials.gov

Database	
ClinicalTrials.gov	
Plattform (potentially incl. Version)	
https://clinicaltrials.gov/ct2/home	
Time period of database	
?–current	
Time limitation	
none	
Standard-searchfilter	
none	
Other limitations	
none	
Date of search	
2019-07-16	
Executed by	
Helge Knüttel	
Peer Review	
no	
Quantity of matches with doublets	
236 = 181 + 47 + 8	
Exportformat of matches	
tsv, xml	
Basic search interface, no filters<br><br>The search had to be separated into three strings because only 250 signs were possible. According to that the quantity of matches was exported individually for every search and there could be doublets/overlapping<br><br>A total of 184 individal matches (NCT Numbers) are include:<br>$ grep --no-filename "^ NCT Number: " kaluza_2738_2019-07-16_ClinicalTrials_s*_records.txt | sort | uniq | wc -l<br>184<br><br>Extraction of doublets has to be done in citavi	

Searchhistory

181 Studies found for: malocclusion OR "posterior occlusion" OR (("class II" OR "class 2") AND occlusion) OR distoclusion OR disto-occlusion OR distocclusion OR "distal occlusion" OR "distal bite" OR retrognath OR retrognathic OR retrognathia OR retrognathism

47 Studies found for: "prominent upper teeth" OR overjet OR "over jet" OR over-jet OR overbite OR "over bite" OR over-bite OR deepbite OR "deep bite" OR deep-bite

8 Studies found for: ((mandible OR mandibular) AND (micrognathia OR micrognathism OR retroposed OR retroposition OR retrused OR retrusion)) OR ((maxilla OR maxillary) AND (prognath OR prognathic OR prognathia OR prognathism OR anteposition OR antepositioned))

Manual search

search in contents of magazines, websites, conferences a.o.

Database	***quantity***	***notes***
MEDLINE	5284	Within doublets 5281individual matches
EMBASE	4548	Within doublets, 4538 individual matches
Cochrane Library:<br>• CDRS (Cochrane Database of Systematic Reviews)<br>• DARE (Database of Abstracts of Reviews of Effect)<br>• CENTRAL (Cochrane Central Register of Controlled Trials)<br>• HTA (Health Technology Assessment Database)<br>• EED (NHS Economic Evaluation Database)	353	
Science Citation Index Expanded	2024	
Google Scholar	200	Only first 200 exported
WHO's International Clinical Trials Registry Platform	312	
ClinicalTrials.gov	236	Within doublets 184 individual matches
Gesamtzahl aus Datenbanksuche	12957	

sources

1. McGowan J, Sampson M, Lefebvre C. An Evidence Based Checklist for the Peer Review of Electronic Search Strategies (PRESS EBC). EBLIP 2010; 5(1):149–54. Available from: URL: http://ejournals.library.ualberta.ca/index.php/EBLIP/article/view/7402.

2. Atkinson KM, Koenka AC, Sanchez CE, Moshontz H, Cooper H. Reporting standards for literature searches and report inclusion criteria: making research syntheses more transparent and easy to replicate. Res. Syn. Meth. 2015; 6(1):87–95.

3. Moher D, Liberati A, Tetzlaff J, Altman DG, The PRISMA Group. Preferred Reporting Items for Systematic Reviews and Meta-Analyses: The PRISMA Statement. PLoS Med 2009 [cited 2013 Feb 22]; 6(7):e1000097.

4. Rader T, Mann M, Stansfield C, Cooper C, Sampson M. Methods for documenting systematic review searches: a discussion of common issues. Res Synth Methods 2014; 5(2):98–115.

5. Shea BJ, Reeves BC, Wells G, Thuku M, Hamel C, Moran J et al. AMSTAR 2: A critical appraisal tool for systematic reviews that include randomised or non-randomised studies of healthcare interventions, or both. BMJ 2017; 358:j4008.

6. Niederstadt C, Droste S. Reporting and presenting information retrieval processes: the need for optimizing common practice in health technology assessment. Int J Technol Assess Health Care 2010; 26(4):450–7.

Appendix 3 – In-/Exclusion Criteria

study characteristics (PICOS)	Inclusion Criteria	Inclusion criteria met?			Localisation (page)
		yes	no	vague	
**Study type S**<br>Requirement for inclusion:<br>1x yes checked, at systematic reviews with meta-analysis 2x yes checked is possible	Systematic Review	☐	☐	☐	
	Meta-Analysis	☐	☐	☐	
	Randomized-controlled trial (RCT)	☐	☐	☐	
	Controlled-clinical study (CCT)	☐	☐	☐	
	Cohort study	☐	☐	☐	
	Case control study	☐	☐	☐	
**Population P**<br>Requirement for inclusion:<br>4x yes checked, which means all 4 criteria have to apply	Class II-anomaly	☐	☐	☐	
	No syndrome	☐	☐	☐	
	No cleft lip and palate	☐	☐	☐	
	No trauma	☐	☐	☐	
**Intervention I**<br>Requirement for inclusion :<br>1x yes checked	(unimax) lower jaw forward	☐	☐	☐	
	(unimax) upper jaw up/backward	☐	☐	☐	
	(bimax) upper jaw up/bachward + lower jaw forward	☐	☐	☐	
**Kontrolle C**<br>Requirement for inclusion:<br>at least 1x yes checked	Other intervention	☐	☐	☐	
	No intervention	☐	☐	☐	
	None, i.e. post OP – post Relapse (longitudinal comparison)	☐	☐	☐	
**Outcome O**<br>Requirement for inclusion:<br>At least 1x yes checked	*Primary:* relapse-extent at least 1 year post OP recorded quantitatively (metric or angular)	☐	☐	☐	
	Secondary: QoL (Quality of life) with validated survey	☐	☐	☐	

INCLUSION ☐	EXCLUSION ☐
